# Breast Milk from Tanzanian Women Has Divergent Effects on Cell-Free and Cell-Associated HIV-1 Infection In Vitro

**DOI:** 10.1371/journal.pone.0043815

**Published:** 2012-08-28

**Authors:** Magdalena A. Lyimo, Matilda Ngarina Mosi, Molly L. Housman, Muhammad Zain-Ul-Abideen, Frederick V. Lee, Alexandra L. Howell, Ruth I. Connor

**Affiliations:** 1 Department of Physiology, The Geisel School of Medicine at Dartmouth, Lebanon, New Hampshire, United States of America; 2 Program in Experimental and Molecular Medicine, The Geisel School of Medicine at Dartmouth, Lebanon, New Hampshire, United States of America; 3 Department of Microbiology and Immunology, The Geisel School of Medicine at Dartmouth, Lebanon, New Hampshire, United States of America; 4 Muhimbili National Hospital and Muhimbili University of Health and Allied Sciences, Dar es Salaam, Tanzania; 5 Veterans Affairs Medical Center, White River Junction, Vermont, United States of America; New York University, United States of America

## Abstract

Transmission of HIV-1 during breastfeeding is a significant source of new pediatric infections in sub-Saharan Africa. Breast milk from HIV-positive mothers contains both cell-free and cell-associated virus; however, the impact of breast milk on HIV-1 infectivity remains poorly understood. In the present study, breast milk was collected from HIV-positive and HIV-negative Tanzanian women attending antenatal clinics in Dar es Salaam. Milk was analyzed for activity *in vitro* against both cell-free and cell-associated HIV-1. Potent inhibition of cell-free R5 and X4 HIV-1 occurred in the presence of milk from all donors regardless of HIV-1 serostatus. Inhibition of cell-free HIV-1 infection positively correlated with milk levels of sialyl-Lewis^X^ from HIV-positive donors. In contrast, milk from 8 of 16 subjects enhanced infection with cell-associated HIV-1 regardless of donor serostatus. Milk from two of these subjects contained high levels of multiple pro-inflammatory cytokines including TNFα, IL-1β, IL-6, IL-8, MIP-1α, MIP-1β, MCP-1 and IP-10, and enhanced cell-associated HIV-1 infection at dilutions as high as 1∶500. These findings indicate that breast milk contains innate factors with divergent activity against cell-free and cell-associated HIV-1 *in vitro*. Enhancement of cell-associated HIV-1 infection by breast milk may be associated with inflammatory conditions in the mother and may contribute to infant infection during breastfeeding.

## Introduction

Transmission of HIV-1 from mother to child during breastfeeding results from the presence of both cell-free virus and HIV-infected cells in the milk [1], [2]. The mechanisms associated with HIV-1 breast milk transmission not well understood; however, the low incidence of infection among most breastfeeding infants of HIV-1 seropositive mothers suggests that HIV-1 transmission is relatively inefficient and supports a protective role for breast milk in preventing viral infection [3]. In clinical studies, components in breast milk, including IL-15 [4], long-chain fatty acids [5] and erythropoietin [6] have been linked to lower rates of post-natal HIV-1 transmission. *In vitro*, breast milk mucin (MUC1) effectively blocks binding and transfer of virus from dendritic cells (DC) to CD4^+^ T cells [7], [8] and inhibits HIV-1 infection [9], [10]. The inhibitory effects of MUC1 are attributed to a rich array of repeating Lewis^X^ motifs, consistent with a direct role for Lewis^X^ in preventing binding of HIV-1 to DC-SIGN [7], [8]. Human milk oligosaccharides and glycans, which are abundant in breast milk, constitute another form of innate immunity against infection with pathogens, including HIV-1 [11]–[13].

In the milieu of breast milk, less is known about the impact of milk factors on cell-associated HIV-1. In contrast to cell-free HIV-1 infection, cell-associated infection arises from direct cell-to-cell transfer of virus to susceptible target cells. Cell-to-cell spread of HIV-1 in culture is significantly more efficient than spread of cell-free virus and involves formation of a transient yet complex virological synapse [14]–[17], which is more difficult to neutralize [15].

Administration of highly active antiretroviral therapy (HAART) to HIV-positive women for the prevention of mother-to-child transmission (PMTCT) during breastfeeding is associated with a rapid decrease in the levels of cell-free HIV-1 RNA in breast milk [18] and a dramatic reduction in HIV transmission [19]–[23]. Transmission rates of less than 2% have been reported in clinical studies of HAART for PMTCT [19]–[21], confirming the effectiveness of this intervention. The reason for residual transmission of HIV-1 in this setting is not clear; however, recent studies have documented transient periods of viremia among women receiving HAART for PMTCT based on intermittent detection of HIV-1 RNA in maternal plasma and breast milk [24]. The presence of a cell-associated reservoir for HIV-1 in breast milk is also suggested by two independent clinical studies documenting the persistence of proviral DNA in breast milk from women receiving HAART during breastfeeding [18], [25]. Taken together, these findings suggest distinct mechanisms that determine the persistence and infectivity of cell-free and cell-associated HIV-1 in breast milk.

In the present study, we sought to evaluate the impact of breast milk on infection of CD4^+^ target cells by cell-free and cell-associated HIV-1 *in vitro*. Our findings suggest that breast milk may have divergent activities against cell-free HIV-1 as compared to cell-associated virus, and this may have implications for understanding the pathogenesis of HIV-1 transmission during breastfeeding and in the setting of HAART for PMTCT.

## Materials and Methods

### Ethics Statement

Protocols for this study were approved by the Institutional Review Boards from the Muhimbili University of Health and Allied Sciences (MUHAS, Dar es Salaam, Tanzania) and Dartmouth College (Hanover, NH). Written informed consent was obtained from all donors prior to collection of milk samples.

### Breast Milk Donors

Breast milk was obtained from 20 women (10 HIV-positive and 10 HIV-negative) enrolled in the antenatal clinic of Muhimbili National Hospital (MNH) in Dar es Salaam, Tanzania, where routine counseling and testing for HIV infection is performed. The HIV-positive milk donors were enrolled in the PMTCT clinic, which is affiliated with the regular antenatal clinic at MNH. Information about methods for preventing mother-to-child transmission of HIV-1 and infant feeding options were made available to HIV-positive milk donors through PMTCT counseling sessions conducted by a nurse at the clinic.

To be eligible for the study, women had to have healthy infants who were breastfeeding without difficulty. Breastfeeding mothers with reported signs and symptoms of mastitis were excluded. A medication history was obtained and included information about administration of single-dose Nevirapine (sdNVP) during delivery and/or ARV treatment received at any time prior to donating breast milk.

### Breast Milk Samples

Milk was collected from either the left or the right breast by manual self-expression. Samples were collected ≥1 hr after the infant last breastfed from the designated side. Milk was collected into sterile plastic tubes and immediately stored and transported at 4°C. Initial processing was performed in the laboratory at MNH within 4 hrs of collection. Whole breast milk was centrifuged at 10,000×*g* for 10 min to separate the cell pellet from the fluid phase of the milk. The cell pellet was washed three times using sterile PBS and stored at −80°C. The lipid layer of the milk was removed manually and the skim milk fraction was aliquoted into 1 mL cryovials and stored at −80°C. The skim milk and cell pellets were shipped on dry ice to The Geisel School of Medicine at Dartmouth for further analyses. Before use in experiments, each aliquot of skim milk was again centrifuged at 10,000×*g* for 5 min to remove any residual lipid and sterile-filtered through a 0.22 micron Millex-GV® filter (Millipore, Billerica, MA). Individual milk samples were not pooled for any experiments.

### HIV-1 Isolates

HIV-1 isolates used for this study included strains with tropism for CCR5 (R5, HIV-1_BaL_) and CXCR4 (X4, HIV-1_HC4_). Virus stocks were propagated in phytohemagglutinin (PHA)-activated peripheral blood mononuclear cells (PBMC) and titered on TZM-bl cells (NIH AIDS Research and Reference Reagent Program, contributed by Dr. John C. Kappes, Dr. Xiaoyun Wu and Tranzyme, Inc.).

### Cell-free HIV-1 Infectivity Assays

The effect of breast milk on cell-free HIV-1 infection was determined by measuring viral Tat-driven activation of the HIV-1 LTR and luciferase expression in TZM-bl cells as previously described [26]. In brief, TZM-bl cells were plated in 96-well plates at a density of 1×10^4^ cells/well in 100 µl of Dulbecco’s Modified Eagle’s Medium (DMEM) containing 1% fetal bovine serum (FBS), antibiotics and amphotericin B. Cell-free HIV-1 (100 TCID_50_) was incubated with five-fold serial dilutions of milk for 15 min prior to addition to TZM-bl cells. Each milk sample was tested in triplicate wells of the plate. The cells were exposed to the mixture of milk and HIV-1 for 24 hrs at 37°C. Following this incubation, the cells were washed and assessed for viability using a methane thiosulfonate (MTS)-based solution (CellTiter 96® Aq_ueous_ One Solution Cell Proliferation Assay, Promega, WI) according to the manufacturer’s instructions. The cells were then lysed and luciferase activity was measured in cell lysates using the Bright-Glo™ Luciferase Assay System (Promega, WI). Luciferase activity was quantified in Relative Light Units (RLU) using a LMaxII^384^ luminometer (Molecular Devices, Sunnyvale, CA). Baseline activation of luciferase expression with media or milk alone (in the absence of added HIV-1) was also determined. Percent (%) inhibition or enhancement of HIV-1 infection was calculated by the following formula: 1-([RLU milk + HIV] – [RLU milk alone])/([RLU media +HIV] – [RLU media alone]) × 100%.

### Cell-associated HIV-1 Infectivity Assays

To assess the effects of breast milk on cell-associated HIV-1 infection, HIV-infected peripheral blood CD4^+^ T lymphocytes were co-cultured with TZM-bl cells in the presence or absence of milk as described [26]. The levels of luciferase activity were then quantified as an indicator of HIV-1 infection of the TZM-bl target cells. In brief, primary CD4^+^ T lymphocytes were first enriched from PBMC and activated for 48 hr with PHA. Cells were then washed and resuspended in fresh media containing 100 U/mL of interleukin-2 (IL-2), followed by infection with HIV-1_BAL_ for 5 days at 37°C prior to use. One day before the experiment, TZM-bl cells were seeded into the wells of a 96-well plate (1×10^4^ cells/well) and allowed to adhere overnight. On the day of the experiment, serial dilutions of milk were added to designated wells of TZM-bl cells, followed by the addition of washed, HIV-infected CD4^+^ T lymphocytes at a final density of 1×10^5^ lymphocytes per well. After 24 hrs, the cells were lysed directly in the wells with Beta-Glo reagent (Promega, Madison WI) and luciferase activity was quantified. Controls included TZM-bl cells co-cultured with uninfected CD4^+^ T lymphocytes in the presence of either media or milk. Additionally, to control for any cell-free HIV-1 released from the infected lymphocytes during co-culture, an equivalent number of HIV-infected CD4^+^ lymphocytes was seeded into wells in the absence of TZM-bl target cells. The supernatant from these wells, containing cell-free virus, was collected after 24 hrs and added to TZM-bl cells. The effect of breast milk on TZM-bl infection by this cell-free virus was also evaluated.

### Multiplex and ELISA Cytokine Assays

The levels of cytokines, chemokines and growth factors in breast milk samples were measured using commercially available multiplex assays (Bio-Rad Laboratories, Hercules, CA) in conjunction with the DartLab Immune Monitoring Core Facility at The Geisel School of Medicine at Dartmouth. Results were quantified based on individual standard curves generated for each cytokine and the data analyzed using Bio-Plex Manager™ software. SDF-1α was measured in breast milk using a commercially available ELISA (R&D Systems, Minneapolis, MN) according to the manufacturer’s instructions.

### Lewis^X^ ELISA

Measurement of Lewis^X^ in breast milk was carried out by standard ELISA. Milk samples were diluted 1∶10 in 0.2 M NaHCO_3_ buffer, pH 9.6. The diluted samples (100 µl/well) were added to ELISA plates (Maxisorp plates, Nunc) and incubated overnight at 4°C. The plates were washed three times with Tris-buffered saline (TBS)/0.05% Tween 20 [TBST]) buffer and blocked with TBS/1% BSA for 30 min at room temperature. After washing with TBST, the plates were incubated with either mouse anti-human Lewis^X^ IgM or mouse anti-human sialyl- Lewis^X^ IgM monoclonal antibodies (Calbiochem, San Diego, CA) for 2 hr at room temperature, followed by washing with TBST and detection with goat anti-mouse IgM-horseradish peroxidase conjugate (Thermo Scientific/Pierce Biotechnology, Rockford, IL). After washing to remove unbound antibodies, the plates were developed using the 1-Step™ Turbo TMB-ELISA reagent (Thermo Scientific/Pierce Biotechnology) at room temperature. The reaction was stopped after 30 min by the addition of 1 M H_2_SO_4_ and the plates were read on an ELISA plate reader at A_450_ nm.

### Quantitative Real-time PCR Assay

Real-time PCR was used to quantify HIV-1 proviral DNA in cell pellets from individual breast milk samples. Total cellular genomic DNA was isolated from milk cell pellets using a Qiagen blood minikit (Qiagen, Valencia CA). DNA samples were then assayed by real-time PCR for HIV-1 proviral DNA using primers and molecular beacons with broad specificity for different HIV-1 genotypes [27]. Conserved sequences within the HIV-1 gag gene were amplified with primers gagF (5′-ATAATCCACCTATCCCAGTAGGAGAAAT-3′ and gagR (5′-TTTGGTCCTTGTCTTATG TCCAGAATG-3′) and detected using the molecular beacon HIV-1/FAM, 5′GCGAGCCTGGGATTAAATAAAATAGTAAGAATGTATAGCGCTCGC-3′ with the quencher DABCYL. Proviral DNA copy number was quantified based on a standard curve generated from 10-fold serial dilution (from 10^8^ to 10^1^ copies) of an HIV-1 plasmid of known copy number. The number of cell equivalents was quantified by amplification of CCR5 sequences using the primers CCR5-590 (5′-CTTCATCATCCTCCTGACAATCG-3′) and CCR5rc890 (5′- GATTCCCGAGTAGCAGATGACC-3′) and the molecular beacon CCR5/FAM, CGAAGCTTGGGTGGTGGCTGTGTTTGCTTCG with the quencher DABCYL. All PCR reactions were carried out in duplicate in a final volume of 50 µl using HotStart-IT Probe qPCR master mix for real-time PCR (USB Corporation, Cleveland OH). Cycling conditions for both CCR5 and HIV quantification were denaturation (95°C, 5 min) followed by 45 cycles of amplification (95°C, 15 sec; 50°C, 30 sec; 72°C, 30 sec) using an Applied Biosystems 7300 Real-Time PCR System.

### Statistical Analysis

GraphPad Prism v.4 (GraphPad Software, Inc, La Jolla, CA) was used to calculate t-tests and Mann-Whitney U tests. A one-way and two-way analysis of variance (ANOVA) was used to perform group comparisons. Student t-test was used to compare means ± standard error of the mean. Mann-Whitney U test was used to compare the median percent inhibition of HIV-1 and concentration of cytokines present in the milk from the HIV-positive and HIV-negative donors. Spearman’s correlations were used to correlate the concentrations of specific cytokines and the inhibitory activity against cell-free HIV-1. A p-value of ≤0.05 was used to indicate significance.

## Results

### Characteristics of Tanzanian Breast Milk Donors

Breast milk was obtained from a total of 20 HIV-positive and HIV-negative mothers enrolled in the antenatal clinic at MNH. The HIV-positive women were in regular attendance at the PMTCT clinic, where they received counseling on strategies for preventing HIV-1 transmission to their infants. Although the HIV-positive women were enrolled in the PMTCT clinic, none reported having taken either sdNVP or any combination of ARV therapy. They acknowledged being offered medication at delivery, but declined to take it.

Women with clinical mastitis were excluded from the study based on physical evaluation and specific questions about the presence of pain, swelling and discharge from the breasts. The characteristics of the milk donors are summarized (Table 1). All milk samples were obtained from women within 42 days post-partum. Among these, colostrum samples were collected within three days post-partum from two HIV-positive and two HIV-negative donors. The average time of breast milk collection post-partum from HIV-positive donors (12.4 days) was less than that of HIV-negative donors (19.3 days); however, this difference was not statistically significant. This difference was likely due to close follow-up of HIV-positive women enrolled in the PMTCT clinic as compared to healthy HIV-negative women who had no medical indication for more frequent follow-up.

**Table 1:** Characteristics of Tanzanian breast milk donors.

	HIV-positive (n = 10)	HIV-negative (n = 10)
Average age of mother (yrs)	27.5 (range 16–40)	26.5 (range 21–37)
PMTCT medication taken	No	N/A
ARV medication taken	No	N/A
Symptomatic breast illness	No	No
Average time of milk collection (days post-partum)	12.4 (range 1–28)	19.3 (range 2–42)

### Breast Milk from HIV-positive and HIV-negative Donors Inhibits Cell-free R5 HIV-1

**Figure 1:**
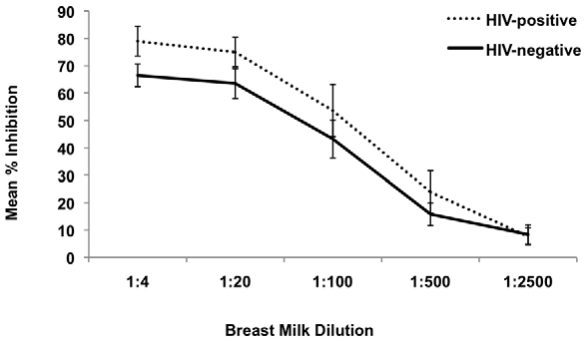
Inhibition of cell-free HIV-1 by breast milk. Mature breast milk from HIV-positive (n = 8) and HIV-negative (n = 8) donors was evaluated for inhibition of cell-free HIV-1 in TZM-bl cell assays. Cells were infected with R5 HIV-1_BaL_ in the presence of five-fold serial dilutions of breast milk. Percent inhibition was calculated relative to control cultures infected with HIV-1_BaL_ in the absence of added breast milk. HIV-inhibitory activity of breast milk from HIV-positive (dotted line) and HIV-negative (solid line) donors is shown (mean ± SD).

To determine if breast milk from HIV-positive women affects cell-free HIV-1 infection *in vitro,* TZM-bl cells were infected with R5 HIV-1_BaL_ in the presence of five-fold serial dilutions (from 1∶4 to 1∶2500) of milk. The effect of mature milk from HIV-positive (n = 8) and HIV-negative (n = 8) donors was evaluated. Significant inhibition of cell-free HIV-1_BaL_ was observed using milk from both HIV-positive donors (dotted line) and HIV-negative donors (solid line) beginning at a 1∶4 dilution (Figure 1). At this dilution, the median percent inhibition of HIV-1 was 85% (range 68%–90.5%) for HIV-positive donors and 64% (range 61%–75.5%) for HIV-negative donors. Although there was a trend toward greater inhibitory activity associated with milk from HIV-positive donors as compared to HIV-negative donors, this difference did not reach statistical significance. The inhibitory activity of breast milk from all donors decreased in direct relation to further dilution of the milk. Only minimal inhibitory activity was observed at a 1∶2500 dilution using milk from either HIV-positive or HIV-negative donors.

Together, these results demonstrate potent HIV-1 inhibitory activity against cell-free R5 HIV-1 in mature milk from both HIV-positive and HIV-negative subjects. Similar results were obtained against cell-free HIV-1_BaL_ using colostrum samples obtained from two HIV-positive and two HIV-negative donors. Compared to mature milk, colostrum had more potent inhibitory activity at a 1∶500 dilution (50% versus 24%). However, the inhibitory activity of colostrum was significantly decreased at a 1∶2500 dilution, a finding that was similar to that observed for mature milk.

Additional experiments were performed to compare the inhibitory activity of breast milk against HIV-1 isolates with difference tropism. Individual milk samples were evaluated against R5-tropic (HIV-1_BaL_) and X4-tropic (HIV-1_HC4_) strains of HIV-1. Among HIV-positive donors, HIV-inhibitory activity in breast milk ranged from 45–96% against HIV-1_BaL,_ and from 25–94% against HIV-1_HC4_. Overall, milk from each donor was able to inhibit both strains of cell-free HIV-1, and there was a significant correlation between the ability to inhibit R5 and X4 strains (R^2^ = 0.596, p = 0.025).

Similar findings were observed using breast milk from HIV-negative donors, demonstrating a significant correlation between the HIV-inhibitory activity against cell-free R5 and X4 HIV-1 (R^2^ = 0.599, p = 0.024). Again, there was a trend toward higher inhibitory activity among milk samples from the HIV-positive donors as compared to HIV-negative donors, similar to our earlier observation (Fig. 1); however, this difference was not statistically significant.

For three HIV-positive donors, sequential breast milk samples were obtained 2 weeks apart. When assayed for HIV-inhibitory activity, milk from 2 of the 3 donors maintained a consistent level of inhibition against cell-free R5 HIV-1_BAL_ between the first and second time points, while the inhibitory activity of milk from the third donor declined (data not shown).

### Levels of Breast Milk MIP-1α, MIP-1β, RANTES and SDF-1α do not Correlate with Cell-free HIV-inhibitory Activity

We next explored whether the HIV-inhibitory activity of breast milk against cell-free HIV-1 was associated with increased concentrations of the ligands for the HIV-1 co-receptors, CCR5 and CXCR4. Levels of MIP-1α, MIP-1β, RANTES and SDF-1α were quantified in the milk of HIV-positive and HIV-negative subjects. The concentration of milk MIP-1α was very low in the majority of samples tested, with mean concentrations of 42.1 pg/ml and 80.1 pg/ml for HIV-positive and HIV-negative donors, respectively. There was no significant difference in the mean level of MIP-1β, (998.3 vs 842.1 pg/ml, Mann-Whitney, p = 0.21) or RANTES (173.2 vs 146.1 pg/ml, Mann-Whitney, p = 0.66), when comparing milk samples from HIV-positive versus HIV-negative donors. The mean concentration of SDF-1α was significantly higher in the milk of HIV-positive donors as compared to HIV-negative donors (1,525 vs 1,189 pg/ml; Mann-Whitney, p = 0.009).

To determine if the levels of breast milk MIP-1α, MIP-1β, RANTES or SDF-1α correlate with inhibitory activity against cell-free HIV-1 *in vitro*, concentrations of each factor were compared to the percent HIV-inhibition for the same sample. Spearman’s correlations were performed for MIP-1α, MIP-1β, RANTES and SDF-1α and the inhibitory activity of all milk samples from both HIV-positive and HIV-negative donors. The results of these comparisons revealed no significant correlation between the level of MIP-1α, MIP-1β, RANTES or SDF-1α in milk and cell-free HIV-inhibitory activity for all donors, independent of HIV-1 serostatus. Additionally, there was no correlation between the concentration of MIP-1α, MIP-1β, RANTES or SDF-1α in milk and the percent inhibitory activity against cell-free HIV-1 when comparing HIV-positive and HIV-negative donors in separate analyses.

### Breast Milk Contains Elevated Levels of Inflammatory Cytokines

**Figure 2:**
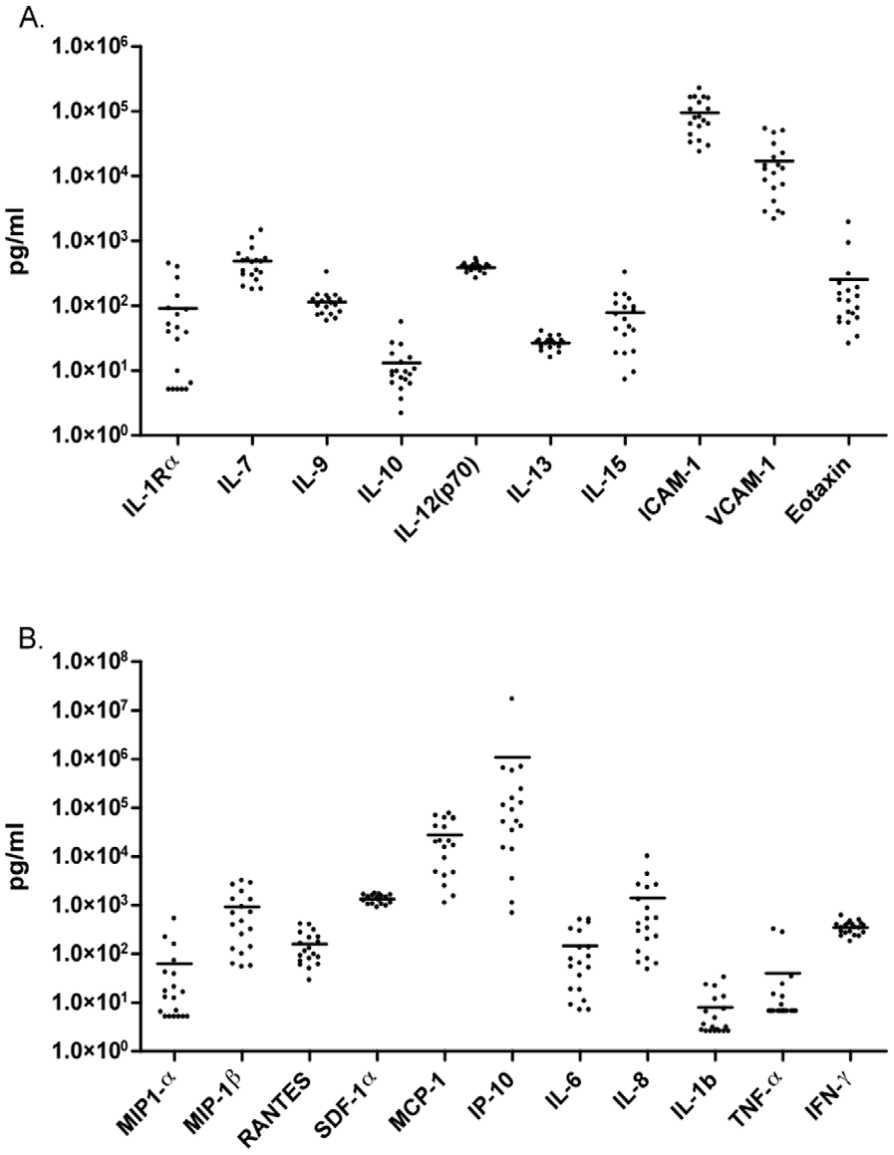
Immune factors in breast milk from Tanzanian donors. The concentrations of multiple immune factors including cytokines, chemokines and growth factors were measured in breast milk samples using a multiplex panel. Data for each factor is expressed as concentration (pg/mL) and is presented for all donors irrespective of HIV serostatus.

Breast milk from both HIV-positive and HIV-negative donors was further evaluated for the presence of an expanded range of cytokines, chemokines and growth factors using a multiplex panel. Among milk samples from all donors, considerable variation was observed in the range of concentrations of the factors measured (Figure 2). In particular, IL-6, IL-8, MIP-1α, MIP-1β, MCP-1 and IP-10 had ≥1 log variation among donors. Of note, subject 002+ (HIV-positive) and subject 002- (HIV-negative) were remarkable for having significantly higher concentrations of multiple inflammatory factors, including TNFα, IL-1β, IL-6, IL-8, MIP-1α, MIP-1β, MCP-1 and IP-10 in their milk as compared to other donors. No significant differences were observed in the levels of individual immune factors among milk samples from HIV-positive and HIV-negative donors, and no significant correlations were found between the level of individual cytokines in milk and inhibition of cell-free HIV-1.

### Sialyl-Lewis^X^ in Breast Milk from HIV-positive Donors Correlates with Inhibition of Cell-free HIV-1

**Figure 3:**
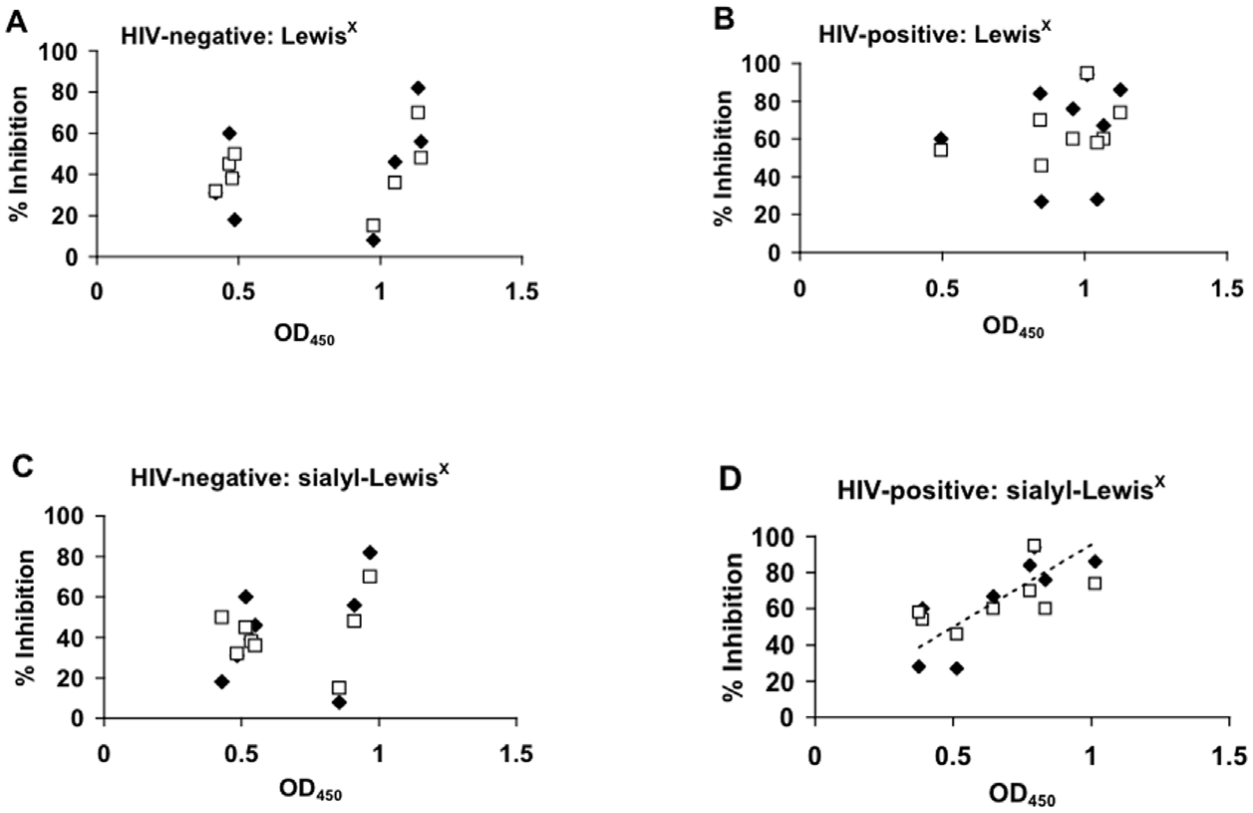
Lewis^X^ and sialyl-Lewis^X^ in breast milk. Levels of Lewis^X^ and sialyl-Lewis^X^ were measured by ELISA in breast milk samples from both HIV-positive and HIV-negative donors. The results were then compared to cell-free HIV-inhibitory activity for the same samples. Data is expressed as the percent inhibition of cell-free R5 (□) and X4 (♦) HIV-1 relative to the levels of either Lewis^X^ or sialyl-Lewis^X^ measured for each sample.

Previous studies have indicated a role for the blood group antigen Lewis^X^ in blocking the interaction between HIV-1gp120 and DC-SIGN expressed on dendritic cells (DC) [8]. Similarly, epithelial-derived MUC1 in breast milk, which contains multiple repeating Lewis^X^ motifs, has been implicated in preventing cell-free HIV-1 transmission to DC and infection of CD4^+^ T cells [8]–[10]. In the present study, levels of Lewis^X^ and sialyl-Lewis^X^ were measured in the breast milk of Tanzanian donors. Results demonstrate the presence of both Lewis^X^ and sialyl-Lewis^X^ in the milk of all donors, with distinct clustering of low and high expression patterns among individual donors (Figure 3). When compared with HIV-inhibitory activity against cell-free R5- or X4-tropic virus, no correlation was found between the levels of milk Lewis^X^ or sialyl-Lewis^X^ among HIV-negative donors or the levels of Lewis^X^ among HIV-positive donors (Fig. 3A–C). However, a significant correlation was observed among HIV-positive donors between the levels of sialyl-Lewis^X^ in milk and cell-free HIV-inhibitory activity (R^2^ = 0.657) (Fig. 3D). These results support a possible role for sialyl-Lewis^X^ in breast milk in preventing cell-free HIV-1 infection of susceptible CD4^+^ target cells.

### Breast Milk Enhances Cell-associated HIV-1 Infection *in vitro*

**Figure 4:**
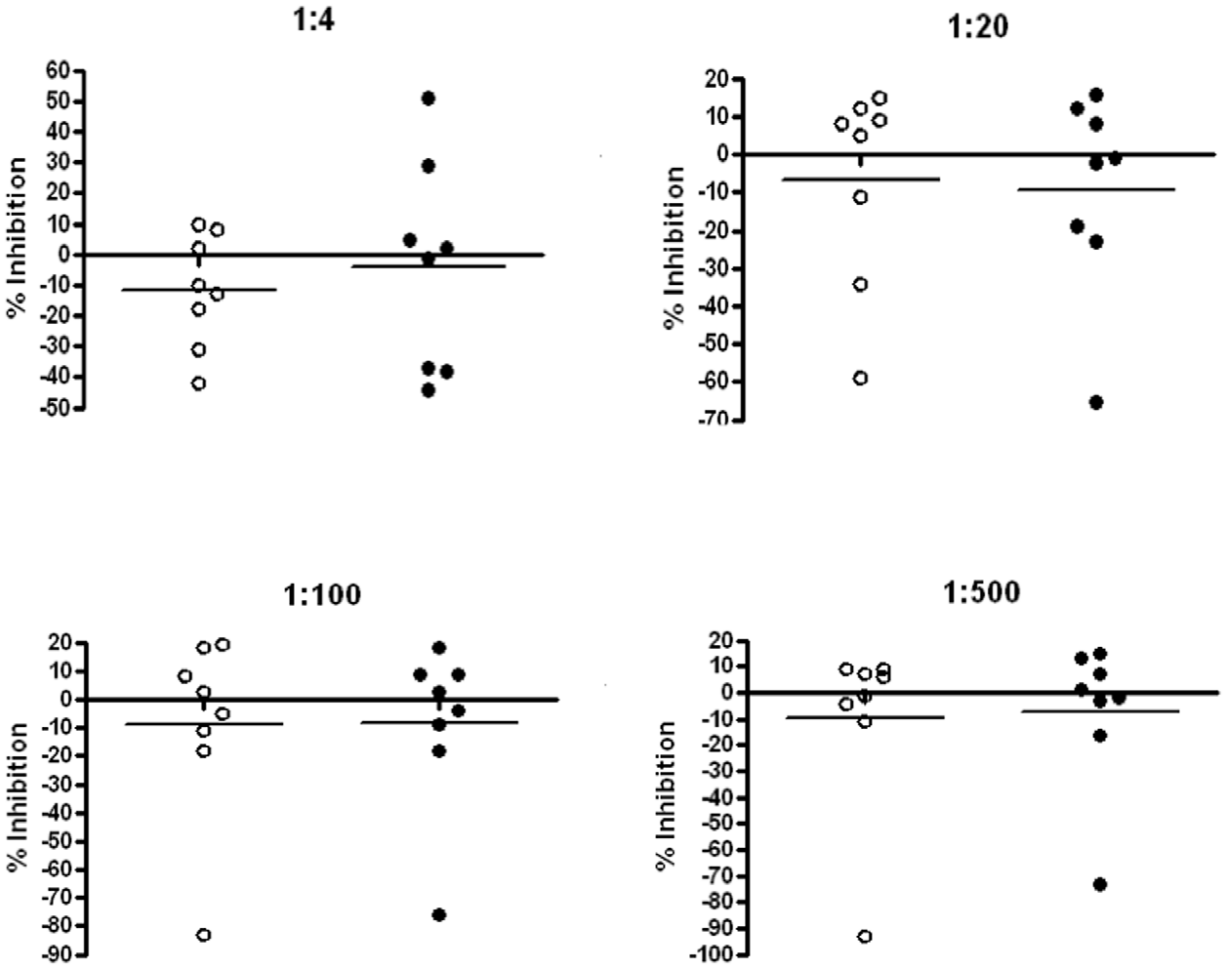
Enhancement of cell-associated HIV-1 infection by breast milk. Breast milk from HIV-positive and HIV-negative donors was evaluated for activity against cell-associated HIV-1 infection in TZM-bl cell assays. Breast milk was tested at 5-fold serial dilutions (1∶4, 1∶20, 1∶100 and 1∶500). Percent inhibition or enhancement of cell-associated R5 HIV-1_BAL_ is shown for breast milk samples from individual HIV-positive (open circles) and HIV-negative (closed circles) donors.

In further experiments, we assessed the ability of breast milk to inhibit cell-associated HIV-1 infection in vitro. The effect of milk at dilutions of 1∶4, 1∶20, 1∶100 and 1∶500 was evaluated in TZM-bl cells co-cultured with HIV-infected primary CD4^+^ T lymphocytes. In contrast to our results using cell-free virus, inhibition of cell-associated HIV-1 was observed with milk from only 2 of 16 donors, and then only at the lowest dilution (1∶4) (Figure 4). Moreover, significant enhancement of cell-associated HIV-1 infection was observed in the presence of milk from both HIV-positive and HIV-negative donors. Even at dilutions as high as 1∶500, milk from two donors, Subject 002+ (HIV-positive) and Subject 002- (HIV-negative), significantly enhanced cell-associated HIV-1 infection (Fig. 4). As noted earlier, milk from these donors had significantly higher levels of multiple inflammatory factors. No correlation was found between the levels of Lewis^X^ or sialyl-Lewis^X^ in milk samples and the observed enhancing activity against cell-associated HIV-1 (data not shown), suggesting that distinct factors influence the impact of breast milk on cell-free versus cell-associated virus.

### Detection of Proviral DNA in Breast Milk Cells

To determine whether enhancement of cell-associated HIV infection by breast milk *in vitro* was associated with the presence of HIV-infected cells *in vivo*, we used quantitative real-time PCR to assess proviral load in breast milk cells isolated from the HIV-1 positive donors in this study. HIV-1 proviral DNA was detected by real-time PCR in 4 of 9 donors tested. Of those donors with detectable proviral DNA in their breast milk cells, 3 of 4 were found to have breast milk that significantly enhanced cell-associated HIV-1 infection *in vitro*.

## Discussion

The results of this study demonstrate significant inhibition of cell-free HIV-1 by breast milk from both HIV-positive and HIV-negative Tanzanian women. Inhibition of cell-free virus was largely independent of viral tropism; however, there was a trend toward higher levels of inhibition of cell-free HIV-1 with milk from HIV-positive as compared to HIV-negative subjects, suggesting both innate and adaptive immune factors may contribute to the overall anti-viral effect of the milk.

Previously, we found that breast milk from HIV-negative donors in the United States potently inhibited cell-free HIV-1 infection *in vitro*, suggesting the presence of innate anti-viral factors at relatively high abundance in breast milk [26]. Here, we confirm the inhibitory effect of breast milk on cell-free HIV-1 using samples obtained from both HIV-positive and HIV-negative women from sub-Saharan Africa. The anti-viral activity against cell-free HIV-1 did not correlate with the presence or levels of individual cytokines, chemokines or growth factors in the milk, including factors that serve as blocking ligands for the HIV-1 co-receptors, CCR5 and CXCR4.

However, a correlation was found between HIV-inhibitory activity and the siaylated form of the blood group antigen Lewis^X^ in the milk of HIV-positive women. Lewis^X^ and MUC1 isolated from breast milk have been shown to inhibit binding of cell-free HIV-1 to DC-SIGN and to prevent transfer of infection to CD4^+^ T cells [8]–[10]. The observed correlation between breast milk inhibitory activity against cell-free HIV-1 and levels of sialyl-Lewis^X^ in milk suggests sialic acid residues may play a key role in blocking cell-free viral infection. The presence of sialic acid motifs at the ends of carbohydrate side chains has been shown to significantly reduce the inherent infectivity of both SIV and HIV-1 [28], [29], while digestion with neuraminindase, which cleaves sialyl moieties, enhances viral infectivity and increases cell-cell syncytium formation *in vitro* [28].

Despite the finding of potent inhibitory activity against cell-free HIV-1, breast milk from the same subjects had no inhibitory effect on cell-associated HIV infection *in vitro*. Rather, a significant proportion of milk from individual donors, including both HIV-positive and HIV-negative subjects, enhanced the level of cell-associated HIV-1 infection. Enhancement did not correlate with levels of sialyl-Lewis^X^ in the milk or with the presence of individual cytokines or chemokines, indicating that distinct factors in breast milk modulate cell-free versus cell-associated HIV-1 infection.

Enhancement of cell-cell interaction and HIV-1 infection in the presence of breast milk may result from up-regulation of cell surface molecules needed for efficient attachment and interaction of HIV-infected milk cells to target cells in the intestinal epithelium or submucosal tissue. Formation of a virological synapse between HIV-infected and uninfected cells is characterized by a tight adhesive junction, involving HIV-1 gp120 binding to CD4, and membrane interaction with adhesion molecules, including ICAM-1 [30]. Breast milk contains high levels of soluble ICAM-1 (sICAM-1), which might inhibit cell-cell adhesion and modulate cell-associated HIV-1 infection. We measured the levels of sICAM-1 in the milk of subjects in this study, and found a range of concentrations (Fig. 2A), but no correlation with inhibition or enhancement of cell-associated HIV-1 infection. Earlier studies reported no effect of blocking antibodies against ICAM-1 in preventing adhesion of HIV-infected lymphocytic cells to epithelial cells [31], [32], suggesting other molecules are critical to the formation of a tight adhesive junction in this setting.

Alternatively, activation of HIV-1 expression from latently infected cells, and/or enhanced release of virions into the synaptic cleft, may be promoted by the presence of stimulatory factors in breast milk. Cell-associated HIV-1 in breast milk is found primarily in infected CD4^+^ T lymphocytes and macrophages [33]–[35]. When stimulated *in vitro*, HIV-infected milk cells secrete higher levels of virus than the comparable population of cells from peripheral blood [35]. Recently, activated milk CD4^+^ T cells from women receiving HAART were shown to produce HIV-1 *in vitro*, suggesting that these cells may constitute a reservoir that is relatively refractive to treatment [36].

Activation of HIV-1 transcription is known to be modulated by immune cytokines [37], and it is possible that high levels of inflammatory factors in breast milk may play a role in enhancing HIV-1 release from infected cells. Among the subjects in this study, markedly elevated concentrations of multiple inflammatory factors were observed in milk from two subjects, and milk from these subjects also enhanced infection with cell-associated HIV-1 at dilutions up to 1∶500 (the highest dilution tested).

Inflammatory conditions in the breast, including mastitis, are known to increase the risk of HIV-1 transmission during breastfeeding [38]–[40]. Mothers in this study were screened at enrollment, and those with clinical signs and symptoms of mastitis were excluded. However, subclinical mastitis may also increase the levels of inflammatory factors in breast milk. Studies by Kantarci, et al. [41] found a positive correlation between subclinical mastitis, increased milk Na/K ratios, and higher numbers of HIV-infected cells in milk from HIV-infected Tanzanian mothers who transmitted HIV-1 to their infants through breastfeeding. While anecdotal, our results demonstrate that *in vitro* exposure of HIV-infected cells to breast milk containing high levels of multiple inflammatory factors can significantly enhance cell-associated HIV-1 infection.

Further studies are needed to determine the mechanisms of enhancement of cell-associated HIV-1 infection by breast milk. Localized and systemic inflammatory responses in the mother may contribute by increasing the levels of proinflammatory factors in the milk. Among HIV-infected mothers, this may represent an increased risk of virus transmission during breastfeeding. Moreover, the failure of breast milk to block cell-associated HIV-1 may be associated with maintenance of a cellular reservoir of virus in breast milk and may contribute to the residual transmission of HIV-1 to breastfeeding infants among women receiving HAART during lactation.
